# Supplementary material for: Profiling Microbial Communities in Idiopathic Granulomatous Mastitis
Source: Int J Mol Sci. 2023 Jan 5;24(2):1042. doi: 10.3390/ijms24021042 (PMC9863225; doi:10.3390/ijms24021042)
Supplement: Supplementary file 1 [file ijms-24-01042-s001.zip › ijms-2096858-supplementary.pdf]

Profiling Microbial Communities in Idiopathic Granulomatous Mastitis

Supplementary materials

Item number	Title	Main text reference	Supplementary materials page number
Supplementary Table S1	Idiopathic granulomatous mastitis (IGM) and lactational mastitis (LM) patients recruited, samples donated, and analysed.	Page 4<br>Page 19	3
Supplementary Table S2	Median and inter-quartile range of alpha-diversity of breast pus and skin microbial populations in patients with idiopathic granulomatous mastitis (IGM) and control patients with lactational mastitis (LM).	Page 11<br>Page 17	4
Supplementary Figure S1	Boxplot of the alpha-diversity of breast pus and skin microbial populations in patients with idiopathic granulomatous mastitis (IGM) and control patients with lactational mastitis (LM). Paired Wilcoxon sign ranked test compares paired IGM pus and skin samples.	Page 11<br>Page 17	5-6
Supplementary Figure S2	Boxplot of the alpha-diversity of breast pus and skin microbial populations in patients with idiopathic granulomatous mastitis (IGM) and control patient with lactational mastitis (LM). Wilcoxon sign ranked test compares (i) IGM pus and LM pus samples; and (ii) IGM skin and LM skin samples.	Page 11	7-8
Supplementary Figure S3	Distribution of relative abundance of *Corynebacterium* genus in breast pus and skin samples from 3 control patients with lactational mastitis (LM).	Page 14	9
Supplementary Figure S4	Relative abundance of *Corynebacterium kroppenstedtii* in breast **(a)** pus and **(b)** skin samples from 21 patients with idiopathic granulomatous mastitis (IGM) and 3 control patients with lactating mastitis (LM) (LM01, LM02, and LM03).	Page 14<br>Page 16	10-11
Supplementary Figure S5	16S rRNA gene map.	Page 15<br>Page 20	12
Supplementary Table S3	Median relative abundance and interquartile range (IQR) of statistically significant species (before and after adjustments, and after correcting for multiple comparisons) in idiopathic granulomatous mastitis (IGM) and lactational mastitis (LM) pus and skin samples. Species were identified from general linear models for determining	Page 16	13

	multivariable association between sample type, covariates and microbial metagenomic features in paired pus and skin samples from patients with IGM.		
Supplementary Figure S6	Scree plot of eigenvalues and multidimensional scaling (MDS) component numbers of the Bray-Curtis distance between breast pus and skin samples from main text Figure 2.	Page 17	14
Supplementary Table S4	Median relative abundance (%) and the interquartile range (IQR) for statistically significant genera (after adjustments and correcting for multiple comparisons) in idiopathic granulomatous mastitis (IGM) and lactational mastitis (LM) pus and skin samples. Genera were identified from general linear models for determining multivariable association between sample type, covariates and microbial metagenomic features in paired pus and skin samples from patients with IGM.	Page 17	15

Supplementary Table S1. Idiopathic granulomatous mastitis (IGM) and lactational mastitis (LM) patients recruited, samples donated, and analysed. IGM: Idiopathic granulomatous mastitis; LM: Lactational mastitis.

Type of mastitis	**IGM**	**LM**
n	26	6
Skin samples	26	6
Pus samples	21	4
Paired skin and pus samples	21	4
Metagenomic analysis	21	3

Supplementary Table S2. Median and inter-quartile range of alpha-diversity of breast pus and skin microbial populations in patients with idiopathic granulomatous mastitis (IGM) and control patients with lactational mastitis (LM), using Shannon and Simpson indices. Equations used for indices calculation can be found in main text Section *4.5 Statistical Analysis*. Shannon index is an information statistic index that measures the number of different genera, and the evenness in distribution of the genera. More unique genera, or more genera evenness increases Shannon index. Simpson index is a dominance index that gives more weight to common and dominant species. Rare species with low abundance is less important in this diversity index. IGM: Idiopathic granulomatous mastitis; LM: Lactational mastitis.

	**Shannon**				**Simpson**			
	**IGM**		**LM**		**IGM**		**LM**	
	**Pus**	**Skin**	**Pus**	**Skin**	**Pus**	**Skin**	**Pus**	**Skin**
25%	1.91	1.46	1.07	1.07	0.75	0.54	0.45	0.45
median	2.65	1.80	1.66	1.66	0.88	0.74	0.73	0.73
75%	2.92	2.36	2.26	2.26	0.92	0.82	0.81	0.81

Supplementary Figure S1. Boxplot of the alpha-diversity of breast pus and skin microbial populations in patients with idiopathic granulomatous mastitis (IGM) and control patients with lactational mastitis (LM), using **(a)** Shannon and **(b)** Simpson indices. Equations used for indices calculation can be found in main text Section *4.5 Statistical Analysis*.

(a) Shannon index is an information statistic index that measures the number of different genera, and the evenness in distribution of the genera. More unique genera, or more genera evenness increases Shannon index.

(b) Simpson index is a dominance index that gives more weight to common and dominant species. Rare species with low abundance is less important in this diversity index.

Paired Wilcoxon sign ranked test found significantly higher diversity in IGM pus samples, compared to the corresponding paired IGM skin sample, for both Shannon ($p = 0.022$) and Simpson ($p = 0.07$) indices. IGM: Idiopathic granulomatous mastitis; LM: Lactational mastitis.

Supplementary Figure S1.

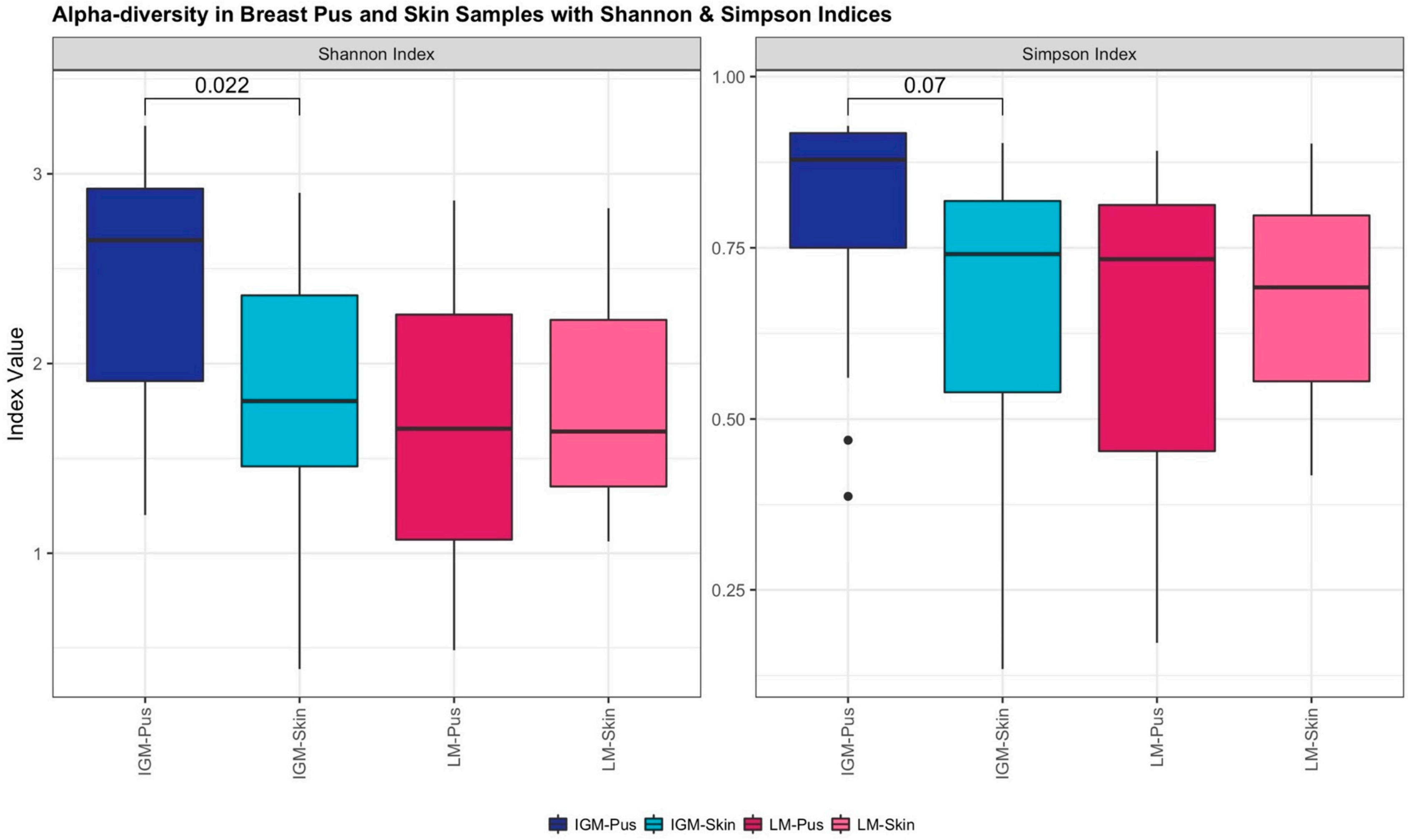

Supplementary Figure S2. Boxplot of the alpha-diversity of breast pus and skin microbial populations in patient with idiopathic granulomatous mastitis (IGM) and control patients with lactational mastitis (LM), using **(a)** Shannon and **(b)** Simpson indices. Equations used for indices calculation can be found in main text Section *4.5 Statistical Analysis*.

(a) Shannon index is an information statistic index that measures the number of different genera, and the evenness in distribution of the genera. More unique genera, or more genera evenness increases Shannon index.

(b) Simpson index is a dominance index that gives more weight to common and dominant species. Rare species with low abundance is less important in this diversity index.

Wilcoxon sign ranked test did not find significant differences between IGM Pus and LM Pus, or IGM Skin and LM Skin samples, in either indices. IGM: Idiopathic granulomatous mastitis; LM: Lactational mastitis.

Supplementary Figure S2.

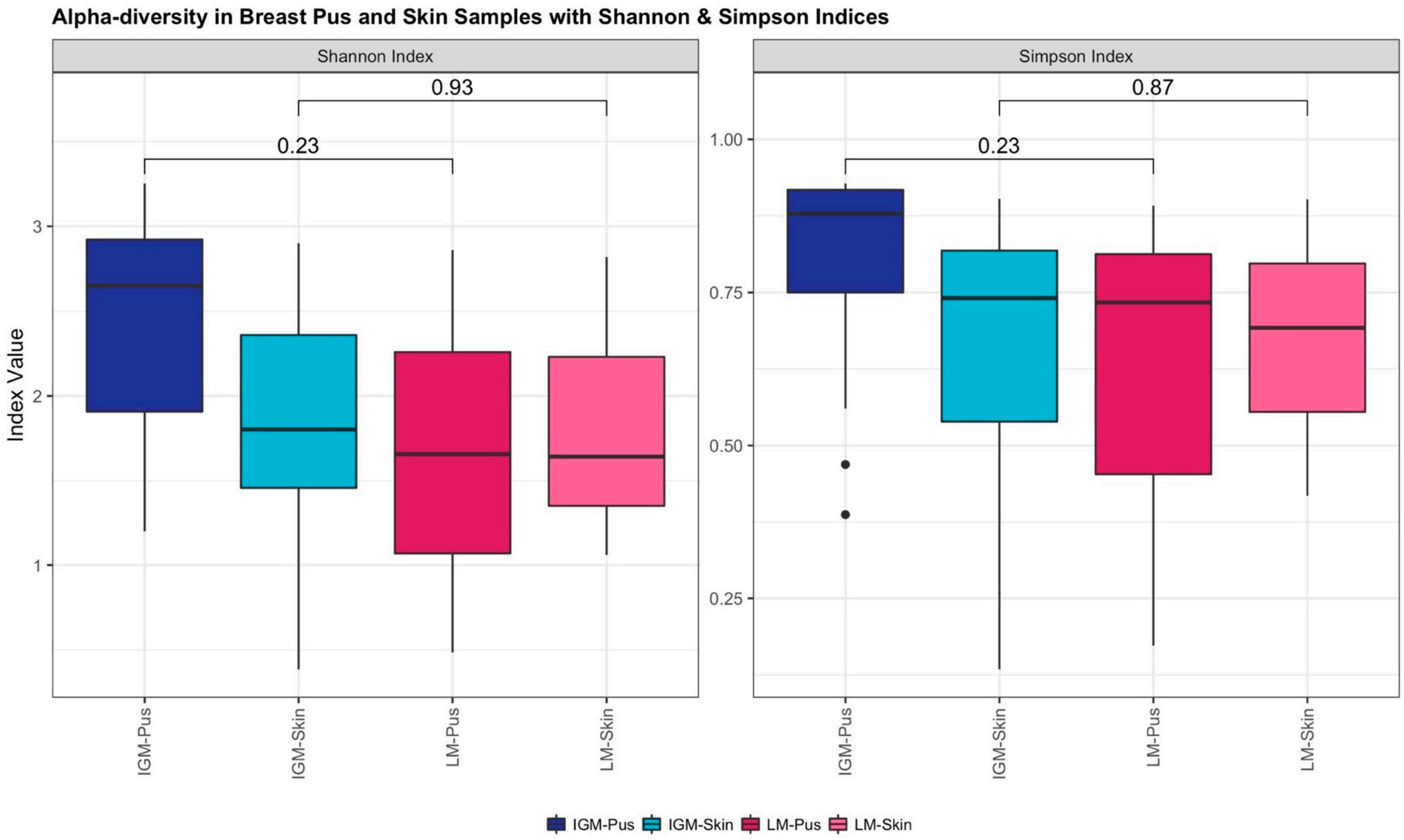

Supplementary Figure S3. Distribution of relative abundance of *Corynebacterium* genus in breast pus and skin samples from 3 control patients lactational mastitis (LM). Median *Corynebacterium* relative abundance in LM pus samples is 1.4% (interquartile range = 0.9-5.1%), compared to 5.8% (interquartile range = 5.6-7.9%) in LM skin samples. Paired Wilcoxon sign ranked test found no significant difference in *Corynebacterium* relative abundance between paired LM skin and pus samples (*p* = 0.25). LM: Lactational mastitis; *p*: *p*-value; Wilcoxon: Wilcoxon paired sign ranked test.

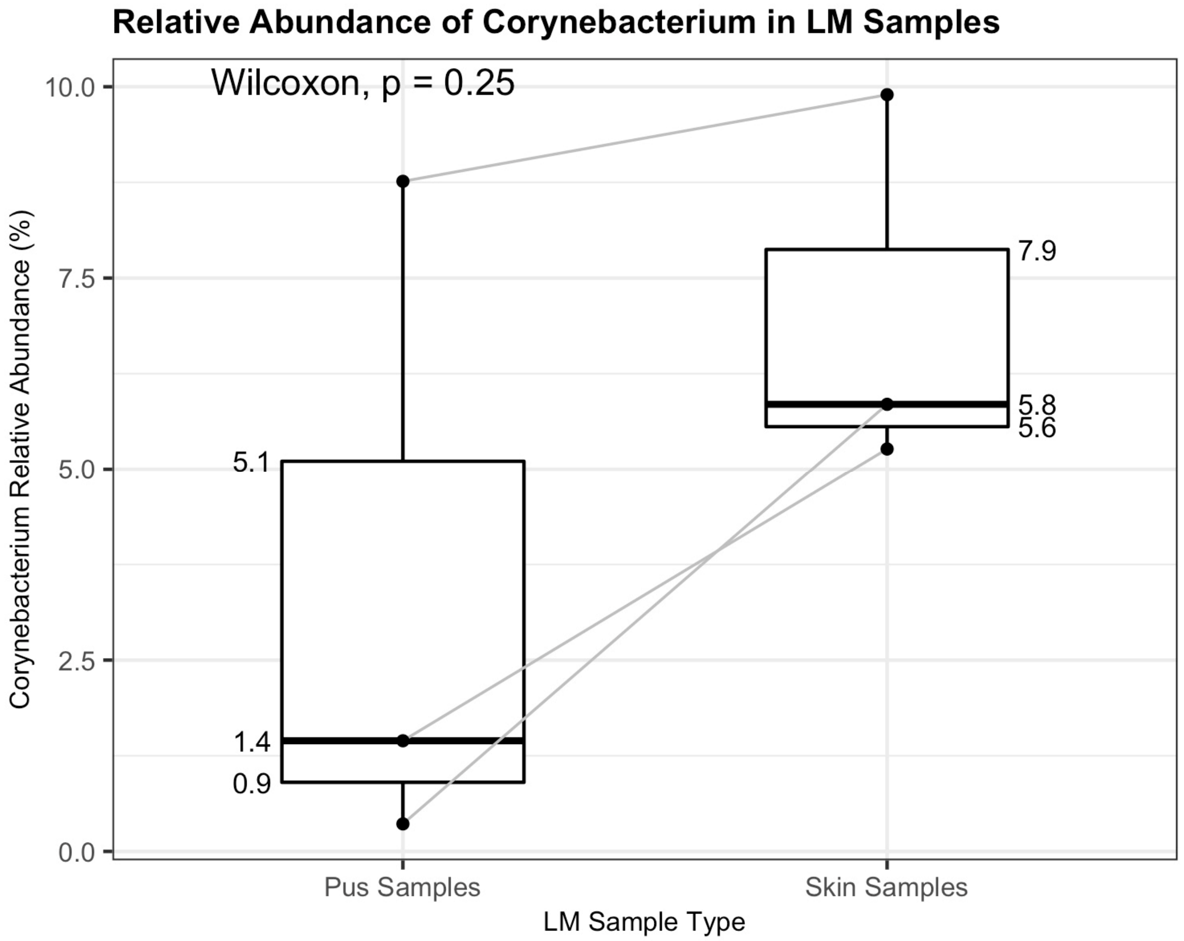

Supplementary Figure S4. Relative abundance of *Corynebacterium kroppenstedtii* in breast **(a)** pus and **(b)** skin samples from 21 patients with idiopathic granulomatous mastitis (IGM) and 3 control patients with lactating mastitis (LM) (LM01, LM02, and LM03). The left-end bars in both panels represent the patients with IGM, and the right-end bars in both panels represent the control patients with LM, with corresponding colours indicating mastitis type shown in the bottom-left shared legend for **(a)** and **(b)**. The percentage of *Corynebacterium kroppenstedtii* relative abundance is indicated above each bar, presented to 1 decimal place. Duration of antibiotic treatment prior to sample collection are presented as symbols above each bar, as four categories: Less than 2 weeks before sample collection, more than 2 weeks before sample collection, missing duration, and no antibiotic treatment. The corresponding symbols are indicated in the bottom-left shared legend for **(a)** and **(b)**.

(a) For the left panel of pus samples, the patients are arranged in decreasing relative abundance of *Corynebacterium kroppenstedtii* in pus samples within mastitis type.

(b) For the right panel of skin samples, the patients are arranged following patient order in **(a)** i.e. decreasing relative abundance of *Corynebacterium kroppenstedtii* in pus samples within mastitis type.

Distribution of relative abundance of *Corynebacterium kroppenstedtii* in breast pus and skin samples from 21 patients with IGM is also displayed in **(a)**. Median *Corynebacterium* relative abundance in IGM pus samples is 2.1% (interquartile range = 0.3-7.0%), compared to 0.1% (interquartile range = 0.0-0.9%) in IGM skin samples. Paired Wilcoxon sign ranked test was significantly higher for *Corynebacterium kroppenstedtii* relative abundance in IGM pus samples than the paired skin samples (p = 0.022).

IGM: Idiopathic granulomatous mastitis; LM: Lactational mastitis; p: p-value; Wilcoxon: Wilcoxon paired sign ranked test.

Supplementary Figure S4.

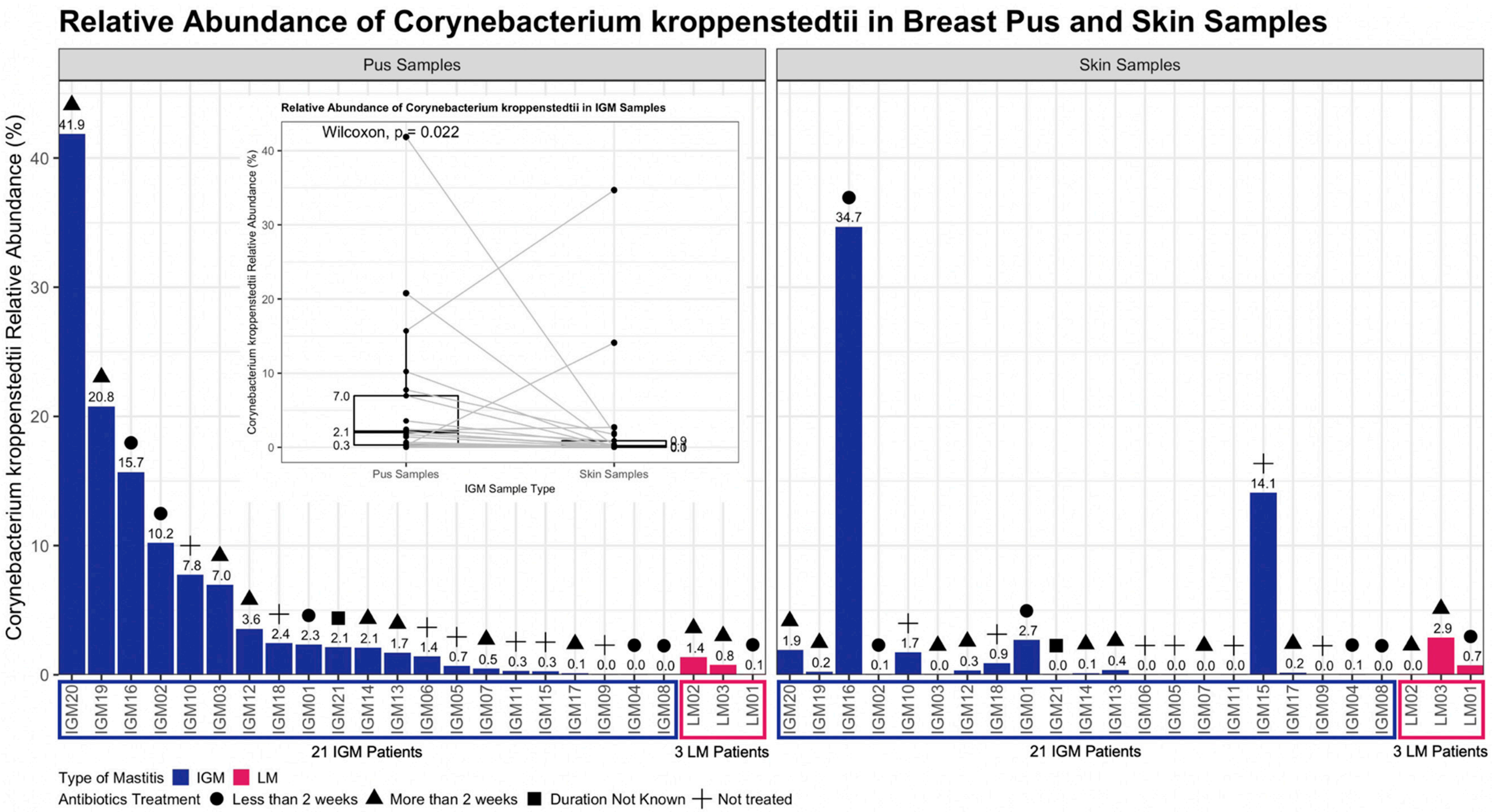

Supplementary Figure S5. 16S rRNA gene map. Variable regions are labelled from 1 to 9, in purple blocks, and conserved regions are in grey. The entire gene is approximately 1,600 base pairs long. Yellow arrows: Forward and reverse primers used in Yu, et al., 2016; Blue arrows: Forward and reverse primers used in our study; primers used and their sequences are shown in main text Table 3. F: Forward primer; R: Reverse primer; V: 16S rRNA gene variable region; bp: Base pairs.

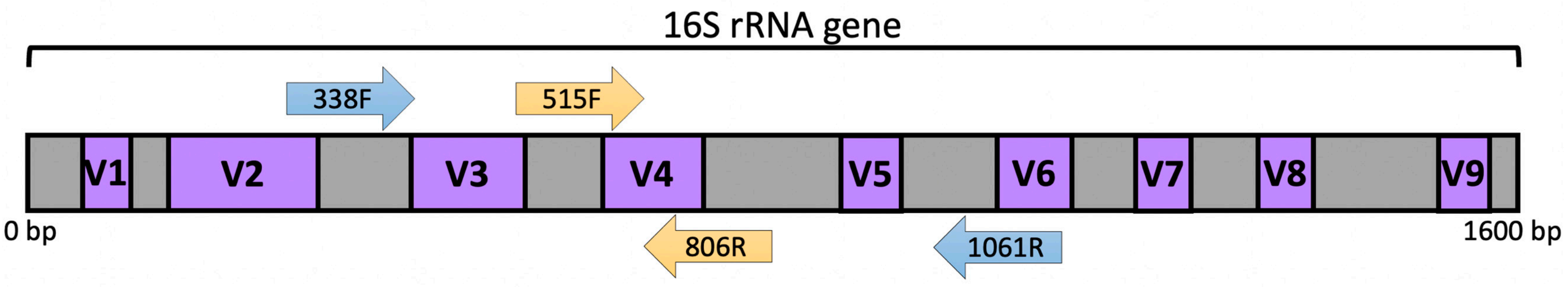

Supplementary Table S3. Median relative abundance (%) and the interquartile range (IQR) for statistically significant species (before and after adjustments, and after correcting for multiple comparisons) in idiopathic granulomatous mastitis (IGM) and lactational mastitis (LM) pus and skin samples. Species were identified from general linear models for determining multivariable association between sample type, covariates and microbial metagenomic features in paired pus and skin samples from patients with IGM. Beta-estimates, standard deviations, crude and adjusted *p*-values, and crude and adjusted q-values are reported in main text Table 2. IQR: Interquartile range; IGM: Idiopathic granulomatous mastitis; LM: Lactational mastitis.

**Species**	**Relative abundance (%) (IQR, %)**			
	**IGM**		**LM**	
	Pus	Skin	Pus	Skin
Acinetobacter schindleri	0.94 (0.04-1.72)	0.00 (0.00-0.00)	0.00 (0.00-0.88)	0.00 (0.00-0.00)
Rothia mucilaginosa	0.27 (0.12-0.53)	0.00 (0.00-0.02)	0.02 (0.01-0.28)	0.00 (0.00-7.12)
Lactobacillus iners	0.00 (0.00-0.00)	0.00 (0.00-0.01)	0.00 (0.00-0.00)	0.00 (0.00-0.00)
Corynebacterium kroppenstedtii	2.10 (0.31-6.96)	0.12 (0.02-0.89)	0.76 (0.42-1.07)	0.72 (0.38-1.79)
Roseomonas mucosa	0.00 (0.00-0.00)	0.03 (0.00-0.13)	0.00 (0.00-0.00)	0.01 (0.00-0.38)
Kocuria rhizophila	0.00 (0.00-0.00)	0.00 (0.00-0.06)	0.00 (0.00-0.00)	0.00 (0.00-0.00)

Supplementary Figure S6. Scree plot of eigenvalues and multidimensional scaling (MDS) component numbers of the Bray-Curtis distance between breast pus and skin samples from main text Figure 2.

(a) Eigenvalues for main text Figure 2(a) MDS of Bray-Curtis distance between breast pus and skin samples for patients with idiopathic granulomatous mastitis (IGM) and control patients with lactational mastitis (LM).

(b) Eigenvalues for main text Figure 2(b) MDS of Bray-Curtis distance between breast pus and skin samples for patients with IGM only.

IGM: Idiopathic granulomatous mastitis; LM: Lactational mastitis.

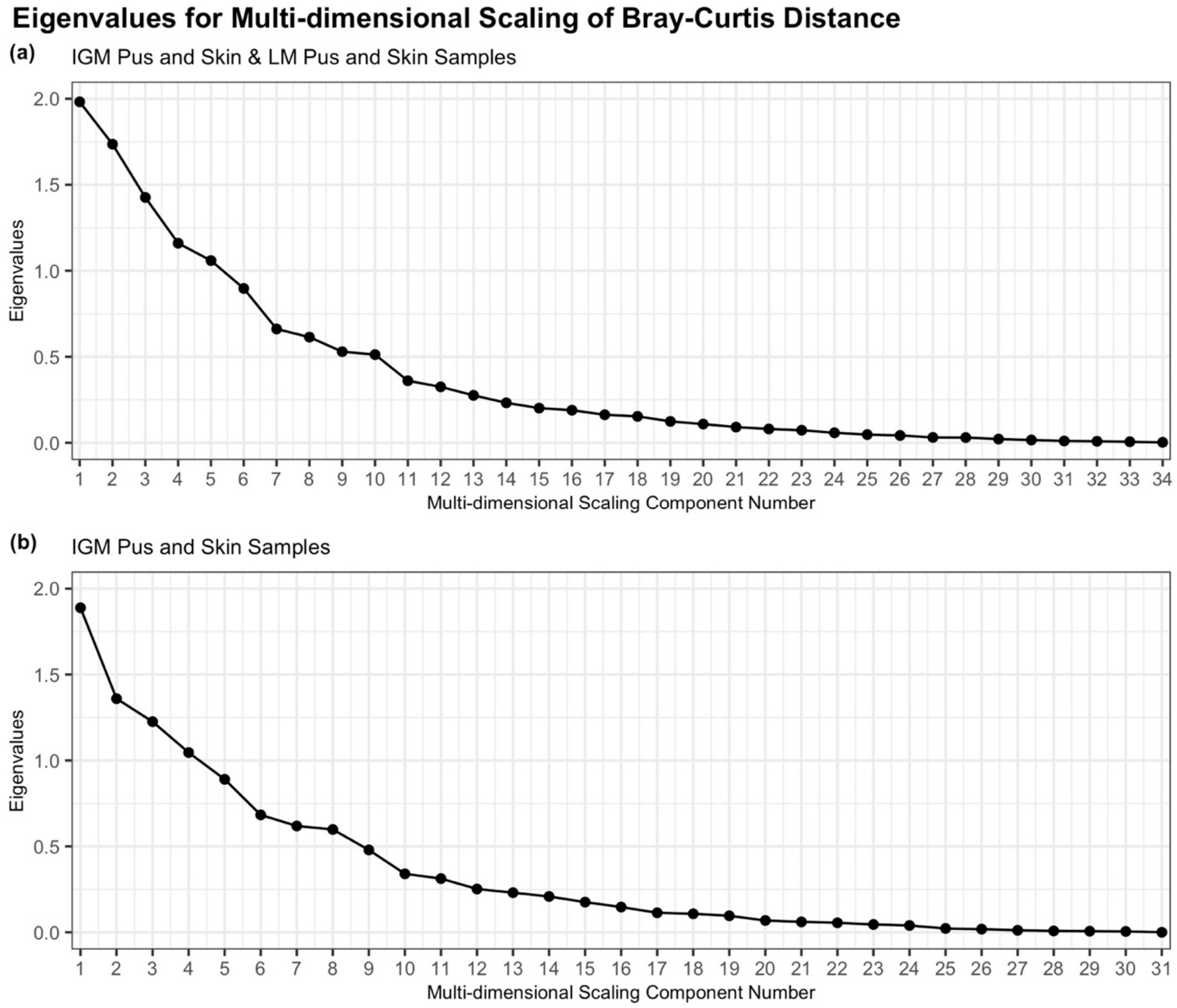

Supplementary Table S4. Median relative abundance (%) and the interquartile range (IQR) for statistically significant genera (after adjustments and correcting for multiple comparisons) in idiopathic granulomatous mastitis (IGM) and lactational mastitis (LM) pus and skin samples. Genera were identified from general linear models for determining multivariable association between sample type, covariates and microbial metagenomic features in paired pus and skin samples from patients with IGM. Beta-estimates, standard deviations, crude and adjusted *p*-values, and crude and adjusted q-values are reported in main text Table 2. IQR: Interquartile range; IGM: Idiopathic granulomatous mastitis; LM: Lactational mastitis.

**Genus**	**Relative abundance (%) (IQR, %)**			
	**IGM**		**LM**	
	Pus	Skin	Pus	Skin
Ochrobactrum	1.75 (1.29-2.81)	0.00 (0.00-0.00)	0.09 (0.04-0.78)	0.00 (0.00-0.00)
Delftia	4.47 (1.85-10.01)	0.01 (0.01-0.01)	0.26 (0.14-2.17)	0.01 (0.01-0.01)
Anaerobacillus	0.60 (0.22-0.98)	0.00 (0.00-0.00)	0.00 (0.00-0.02)	0.00 (0.00-0.00)
Gordonia	0.69 (0.32-1.06)	0.02 (0.01-0.05)	0.05 (0.03-0.55)	0.08 (0.04-0.18)
Methylobacterium	0.00 (0.00-0.00)	0.02 (0.00-0.04)	0.00 (0.00-0.00)	0.01 (0.01-0.01)
Fusobacterium	0.92 (0.35-1.43)	0.01 (0.00-0.03)	0.03 (0.03-1.53)	0.01 (0.01-0.02)
Sphingobium	0.00 (0.00-0.00)	0.00 (0.00-0.02)	0.00 (0.00-0.08)	0.00 (0.00-0.00)
Alkanindiges	0.29 (0.02-0.43)	0.00 (0.00-0.01)	0.02 (0.01-0.19)	0.00 (0.00-0.00)
Streptococcus	3.51 (1.76-5.09)	0.23 (0.14-0.58)	3.95 (2.25-5.69)	8.94 (7.06-29.98)
Achromobacter	0.00 (0.00-0.00)	0.02 (0.00-0.10)	0.00 (0.00-0.00)	0.02 (0.01-0.03)
Capnocytophaga	0.00 (0.00-0.00)	0.00 (0.00-0.03)	0.00 (0.00-0.00)	0.01 (0.01-0.01)
Mycobacterium	0.00 (0.00-0.00)	0.04 (0.01-0.08)	0.00 (0.00-0.00)	0.08 (0.05-0.18)
Novosphingobium	0.00 (0.00-0.00)	0.01 (0.00-0.01)	0.00 (0.00-0.00)	0.01 (0.01-0.07)
Peptoniphilus	0.45 (0.31-0.68)	0.02 (0.01-0.11)	0.02 (0.01-0.33)	0.01 (0.00-0.01)
Rothia	0.29 (0.12-0.53)	0.02 (0.00-0.03)	0.02 (0.01-0.28)	0.03 (0.02-7.13)
Finegoldia	0.94 (0.39-1.13)	0.07 (0.02-0.10)	0.03 (0.02-0.62)	0.01 (0.01-0.02)
Burkholderia	0.00 (0.00-0.00)	0.01 (0.00-0.04)	0.00 (0.00-0.11)	0.00 (0.00-0.01)
Roseomonas	0.00 (0.00-0.00)	0.03 (0.00-0.13)	0.00 (0.00-0.00)	0.01 (0.00-0.38)
Anaerococcus	0.69 (0.40-0.92)	0.06 (0.02-0.19)	0.17 (0.10-0.48)	0.05 (0.02-0.13)
Agrobacterium	0.00 (0.00-0.00)	0.01 (0.01-0.07)	0.05 (0.03-0.06)	0.01 (0.00-0.04)
Hydrogenophaga	0.00 (0.00-0.00)	0.00 (0.00-0.02)	0.00 (0.00-0.00)	0.00 (0.00-0.00)
Peptostreptococcus	0.00 (0.00-0.00)	0.00 (0.00-0.01)	0.00 (0.00-0.00)	0.00 (0.00-0.00)
Dermabacter	0.00 (0.00-0.00)	0.03 (0.00-0.07)	0.00 (0.00-0.00)	0.05 (0.03-0.08)
Kocuria	0.00 (0.00-0.00)	0.03 (0.00-0.07)	0.00 (0.00-0.00)	0.00 (0.00-0.00)
